# Thiol/Disulfide Homeostasis as a New Oxidative Stress Marker in Patients with COPD

**DOI:** 10.3390/diagnostics15202584

**Published:** 2025-10-13

**Authors:** Selen Karaoğlanoğlu, Hüseyin Erdal

**Affiliations:** 1 Department of Pulmonology, School of Medicine, Ordu University, 52200 Ordu, Türkiye; 2 Department of Medical Genetics, School of Medicine, Aksaray University, 68100 Aksaray, Türkiye; huseyinerdal@aksaray.edu.tr

**Keywords:** COPD, oxidative stress, thiol-disulfide homeostasis

## Abstract

**Background and Objectives**: Chronic Obstructive Pulmonary Disease (COPD) is characterized by chronic inflammation and an imbalance between oxidative and antioxidative mechanisms. The aim of this study was to investigate oxidative balance and dynamic thiol/disulfide homeostasis in patients with COPD. **Materials and Methods**: A total of 68 participants, including 34 COPD patients and 34 healthy controls, were enrolled. Demographic characteristics, smoking status, comorbidities, inflammatory and biochemical parameters, and oxidative stress (OS) markers were recorded. Pulmonary function tests were performed, and COPD patients were classified according to GOLD stages. Correlation, ROC, and multivariate logistic regression analyses were conducted to evaluate the relationships between OS, disease severity, and potential confounders. **Results**: Among all participants, smoking and comorbidities were significantly more frequent in COPD patients compared to controls. COPD was associated with increased inflammatory and OS markers, characterized by reduced total and native thiol and total antioxidant status (TAS) levels, alongside elevated disulfide, total oxidant status (TOS), and oxidative stress index (OSI). ROC analysis showed strong diagnostic accuracy for thiol parameters, particularly total and native thiol. Thiol depletion and elevated OS were more pronounced with advancing GOLD stage. In multivariate analysis, smoking status was independently associated with COPD. **Conclusions**: Thiol/disulfide imbalance and OS are evident in COPD, suggesting potential clinical relevance for disease evaluation. However, larger studies are needed to confirm their role as reliable biomarkers or therapeutic targets.

## 1. Introduction

Chronic Obstructive Pulmonary Disease (COPD) is a progressive and debilitating respiratory disorder primarily characterized by chronic inflammation of the airways and persistent airflow limitation [1]. This airflow obstruction, which represents one of the hallmark features of the disease, is both common and potentially preventable. It typically develops as a consequence of prolonged and repeated exposure to harmful particles or toxic gases, such as those found in cigarette smoke, occupational dust, and environmental pollutants [2]. These noxious agents induce oxidative stress (OS) and inflammatory responses within the lungs, leading to structural damage of the airways and alveolar destruction [3]. Over time, these pathological changes contribute to irreversible airflow restriction, reduced gas exchange, and declining pulmonary function, ultimately impairing patients’ quality of life and increasing the risk of exacerbations and comorbidities [4,5].

The pathophysiology of COPD involves chronic inflammation and structural changes in the lungs resulting from prolonged exposure to harmful particles [6]. This exposure triggers an abnormal inflammatory response in the airways, alveoli, and pulmonary vasculature. It is thought to be significantly influenced by OS, which arises from an imbalance between the generation of reactive oxygen species (ROS) and the efficiency of antioxidant defense mechanisms [7]. Cellular injury may result from ROS impacts on a number of cellular functions, including DNA damage, protein oxidation, and lipid peroxidation [8]. Cigarette smoke, air pollution, and mediators released by inflammatory cells are the main causes of elevated OS in COPD. ROS production can be increased and antioxidant defense systems suppressed by these factors [9].

A central feature in the progression of this disease is OS, which arises from an imbalance between ROS and the antioxidant defense system [10]. OS refers to a state in which the production of ROS exceeds the capacity of the body’s antioxidant defense systems to neutralize them. It has been widely reported in the literature that OS plays a pivotal role in the mechanisms underlying several diseases [10,11,12,13,14,15].

In recent years, thiol–disulfide homeostasis has emerged as a sensitive and dynamic marker reflecting the oxidative status of biological systems [16]. Thiol compounds are organic molecules that include a sulfhydryl (-SH) functional group, consisting of sulfur and hydrogen atoms. Due to the high reactivity of this group, thiols are particularly prone to oxidation. In the presence of OS, thiol groups are oxidized to form disulfide bonds. These disulfide bonds can subsequently be reduced back to thiols through the action of reducing agents, thereby maintaining the dynamic equilibrium between thiol and disulfide forms which is crucial for maintaining protein structure, enzyme function, and intracellular signaling [17,18]. The thiol–disulfide balance can be upset in chronic inflammatory diseases like COPD, which increases OS and exacerbates inflammation [19,20]. This study aims to show both dynamic thiol–disulfide balance and OS levels in patients with COPD.

## 2. Materials and Methods

### 2.1. Study Design

This prospective observational study was conducted with 34 patients (6 women and 28 men) who presented to the Chest Diseases outpatient clinic of Ordu University Training and Research Hospital between May 2023 and November 2023. The study protocol was approved by the institutional ethics committee and complied with the ethical standards of the Declaration of Helsinki. Written informed consent was obtained from all participants prior to enrollment.

#### Inclusion and Exclusion Criterias

Patients were eligible for inclusion if they had a confirmed diagnosis of COPD established by a pulmonologist in accordance with the GOLD criteria, provided informed consent to participate in the study, were in a stable phase of the disease without evidence of acute exacerbation, and had not used inhaled or systemic corticosteroids or received antioxidant therapy within the preceding three months. Patients were excluded if they declined to participate in the study, had an unconfirmed diagnosis of COPD, presented with clinical signs of an acute exacerbation, or were currently using inhaled or systemic corticosteroids and antioxidant therapy.

### 2.2. Evaluation of Dynamic Thiol–Disulfide Levels

Native and total thiol concentrations were determined using an automated spectrophotometric technique, as outlined in a prior study (Rel Assay Diagnostics, Türkiye) [21]. In summary, thiol groups were initially reduced using sodium borohydride. To prevent interference with DTNB, any excess sodium borohydride was neutralized and eliminated from the reaction medium using formaldehyde. The concentration of disulfides was calculated by subtracting the native thiol value from the total thiol value and dividing the result by two.

### 2.3. Data Collection

The patients’ hemogram, CRP (C-reactive protein), urea, creatinine, ALT, AST, LDH, native and total thiol, and disulfide levels were measured. For hematological measurements (Hemoglobin, neutrophils, lymphocytes, platelets, monocytes, eosinophils, MPV), approximately 2 cc of blood was drawn from the antecubital vein into EDTA-K2 anticoagulant-containing (purple-capped) hemogram tubes. The tubes were gently inverted (not shaken) and analyzed using a Sysmex XN 1000 (Sysmex Corporation, Kobe, Japan) automated hematology analyzer.

For the analysis of CRP, urea, creatinine, ALT, AST, and LDH parameters, approximately 8 cc of blood was collected from the antecubital vein into biochemistry tubes without anticoagulant. The blood samples were centrifuged at 3000 rpm for 10 min and analyzed using a cobas 8000 c 702 (Roche Diagnostics, Mannheim, Germany) automated biochemistry analyzer. Subsequently, serum samples were aliquoted and stored in Eppendorf tubes at −80 °C until the time of analysis for native and total thiol levels.

### 2.4. Statistical Analysis

Statistical analyses were performed using the MedCalc statistical software package (version 20.009; Ostend, Belgium). Power analysis was performed using the G-Power (v3.1.2) program to determine sample sizes. The study’s power is expressed as 1-beta (Beta = Type II error probability) and has 80% power. According to Cohen’s effect size coefficients, assuming that the evaluations between two independent groups will have a large effect size (d = 0.70), it was calculated that at least 34 participants are required for each group to achieve 80% power at an alpha level of 0.05.

The Shapiro–Wilk test was used to determine whether the data were normally distributed. Categorical data are described as frequencies and percentages. For the comparison of numerical data, the Independent Samples *t*-test was used for normally distributed groups, while the Mann–Whitney U test was used for non-normally distributed groups. In the pairwise comparison tables, data following a normal distribution are presented as mean and standard deviation (SD), while data not following a normal distribution are presented as median (25th percentile–75th percentile). Pearson correlation was used for groups showing normal distribution, and Spearman correlation was used for non-conforming groups. Categorical variables were described as frequencies and percentages. The Chi-square test was used to evaluate categorical variables.

Receiver Operating Characteristic (ROC) analysis was conducted to assess and compare the diagnostic performance of laboratory parameters and OS markers. The Youden J index was used to determine the optimal cutoff value, and the corresponding sensitivity, specificity, positive predictive value (PPV), negative predictive value (NPV), and area under the curve (AUC) with 95% confidence intervals (CIs) were reported. Logistic regression was used to evaluate risk factors associated with COPD status. Odds ratios (ORs) for the parameters and their corresponding 95% confidence intervals (CIs) were reported. A *p*-value of <0.05 was considered statistically significant in the interpretation of the results.

### 2.5. Ethical Considerations

This study received approval from the following institutional ethics committee: Ethics Committee of Non-Interventional Clinical Research of Ordu University (Date: 31 March 2023, Decision No: 2023/89). This study complied with the principles outlined in the Declaration of Helsinki.

## 3. Results

A total of 68 individuals were included in this study, consisting of 34 patients with COPD (6 women and 28 men) and 34 controls (6 women and 28 men). Smoking prevalence was significantly higher in the COPD group (88.2%) compared to the control group (35.3%). Regarding comorbidities, the majority of controls had no comorbidities (58.8%), whereas only 18.4% of COPD patients had no comorbidities. Coronary artery disease and hypertension were similarly distributed between the groups, while diabetes and hyperlipidemia were observed only in the COPD group. Atrial fibrillation was rare, with only one patient in the COPD group. The COPD group was classified according to GOLD stages: 34.4% in Stage 1, 26.5% in Stage 2, 26.5% in Stage 3, and 14.7% in Stage 4 (Table 1).

There were no significant differences between the two groups in terms of gender, age, or body mass index (BMI) (*p* = 1.000, *p* = 0.476, and *p* = 0.578, respectively). In the COPD group, WBC, neutrophil (10^3^/μL), monocyte (10^3^/μL), and MPV (fL) levels were found to be significantly higher compared to the control group (*p* = 0.017, *p* = 0.000, *p* < 0.0001, and *p* = 0.002, respectively). Eosinophil (10^3^/μL) levels were significantly lower in the COPD group than in the control group (*p* = 0.019).

Although there were no statistically significant differences in lymphocyte (10^3^/μL), platelet (10^3^/μL), and hemoglobin (g/dL) levels between the two groups, these parameters were found to be lower in the COPD group (*p* = 0.523, *p* = 0.956, and *p* = 0.446, respectively). Among the biochemical parameters, CRP (mg/L) and urea (mg/dL) levels were higher in the COPD group, though not statistically significant (*p* = 0.229 and *p* = 0.082, respectively), while creatinine (mg/dL) levels were significantly lower (*p* = 0.015). ALT (IU/L) levels were significantly lower in the COPD group, whereas AST (IU/L) and LDH (IU/L) levels were significantly higher (*p* = 0.000, *p* = 0.025, and *p* = 0.000, respectively) (Table 2).

In the comparison of OS parameters between the patient and control groups, total thiol (µmol/L), native thiol (µmol/L), native thiol/total thiol ratio, and TAS levels were found to be significantly lower in the COPD group compared to the control group (*p* < 0.0001, *p* < 0.0001, *p* < 0.0001, and *p* < 0.0001, respectively). On the other hand, disulfide, disulfide/native thiol ratio, disulfide/total thiol ratio, TOS level, and OSI level were significantly higher in the COPD group (*p* = 0.048, *p* = 0.000, *p* < 0.0001, *p* < 0.0001, and *p* < 0.0001, respectively) (Table 3).

When OS parameters were analyzed according to GOLD stages, no statistically significant differences were observed, indicating that disease severity was not clearly associated with thiol–disulfide balance in this cohort (Table 4).

Correlation analysis between pulmonary function test results and OS markers is presented in Table 5. No statistically significant correlations were observed between pulmonary function parameters and OS markers in COPD patients (Table 5).

In the ROC analysis conducted to evaluate the diagnostic performance of OS parameters in relation to COPD, the optimal cutoff value for total thiol (µmol/L) was determined to be 425.2, with 94% sensitivity and 91% specificity. For native thiol (µmol/L), the optimal cutoff value was found to be 394.5, with 97% sensitivity and 94% specificity. For disulfide, the optimal cutoff value was determined to be 15.5, with 91% sensitivity and 44% specificity (Table 6).

**Figure 1:**
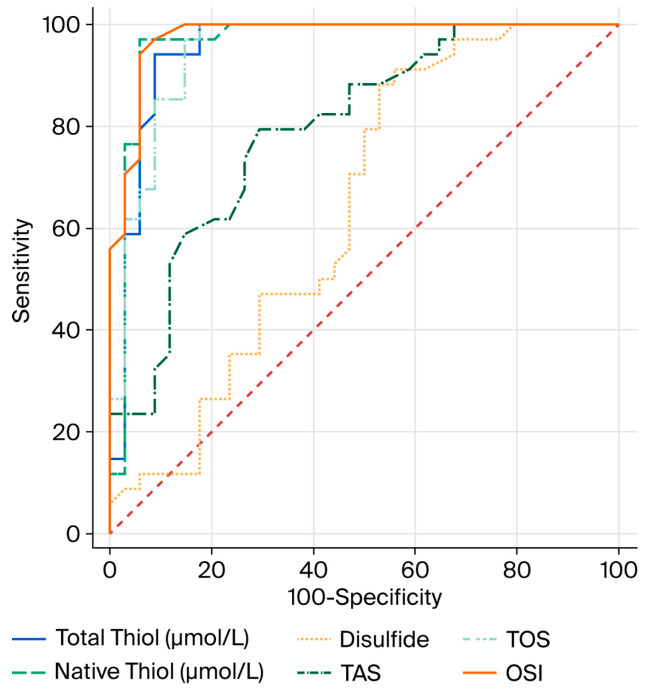
ROC curve comparison of oxidative stress parameters for COPD.

The AUC values of total thiol (µmol/L), native thiol (µmol/L) and disulfide, which are OS parameters, were found to be 0.952, 0.962, and 0.640, respectively (Figure 1).

We performed a multivariate logistic regression analysis to control for potential confounders including gender, smoking status, comorbidities, age, and BMI. According to the analysis, smoking status was independently associated with COPD (OR = 0.062, 95% CI: 0.01–0.32, *p* = 0.001), while other variables did not reach statistical significance (Table 7).

## 4. Discussion

In this study, OS levels and thiol/disulfide balance in patients with COPD were evaluated by comparing them with a control group. Our findings revealed a marked increase in OS and a weakened antioxidant defense system in COPD patients. Specifically, the significantly lower levels of total thiol, native thiol, and TAS, along with significantly higher levels of disulfide, disulfide/native thiol ratio, disulfide/total thiol ratio, TOS, and OSI in the COPD group, indicate that OS imbalance plays a significant role in the pathogenesis of COPD. These results are consistent with previous studies [22].

Thiol groups are important antioxidants capable of neutralizing reactive oxygen species (ROS); therefore, a decrease in thiol levels suggests a weakening of the antioxidant defense system [23]. The increase in disulfide levels may be due to the oxidation of thiol groups as a result of OS [24]. Furthermore, the high sensitivity and specificity values obtained from the ROC analyses of total and native thiol levels indicate that these parameters may serve as potential biomarkers for the diagnosis of COPD.

These findings are important not only for identifying potential biomarkers, but also for providing information about the future development and application of antioxidant-based therapies. Thiol-targeted therapies may exhibit different effects depending on the approach used. Sensitive redox modulation aims to restore redox balance in a controlled and region-specific manner by targeting areas or pathways with excessive OS, thereby minimizing potential side effects and preventing disruption of physiological ROS signaling. In contrast, general antioxidant supplementation provides a systemic increase in antioxidants, which may not selectively address pathological OS and could potentially interfere with normal cellular signaling. Therefore, strategy selection is crucial, and targeted approaches may offer greater efficacy and safety in modulating OS in COPD [25].

In the study by Caliskan et al. [26], they examined the relationship between mortality and thiol disulfide homeostasis in COPD patients with hypercapnic respiratory failure, and it was reported that native and total thiol levels were significantly lower than in the control group, whereas the mean disulfide (Ds)/TT and Ds/NT values were significantly higher. However, no significant difference was detected between the two groups in terms of disulfide levels. They concluded that thiol/disulfide parameters can be used in the diagnosis of hypercapnic respiratory failure and in the prediction and improvement of prognosis in emergency departments and hospitals.

In another study conducted by Eroğlu et al. [27], it was reported that thiol levels were significantly lower in COPD patients compared to the control group. In addition, Ischemia-Modified Albumin (IMA), disulphide/native thiol, disulphide/total thiol and native thiol/total thiol ratios were significantly higher. They hypothesized that thiol disulfide levels and IMA can be used to monitor OS emerging in COPD.

In the study conducted by Solak et al. [28] on patients who smoked, they reported that thiol levels were significantly lower whereas disulfide levels were significantly higher than in the control group. They stated that the reason for this was that smoking increased OS and, as a result, thiol groups were converted into disulfide structures.

In our study, hematological and biochemical parameters were also evaluated. The significantly higher levels of WBC, neutrophils, monocytes, and MPV in the COPD group compared to the control group indicate an increase in systemic inflammation [7]. The significantly lower eosinophil levels suggest a variable eosinophilic profile in COPD. The elevations in AST and LDH levels can be interpreted as indicators of cellular damage [29]. Although some biochemical parameters such as creatinine, ALT, AST, and LDH demonstrated statistically significant differences between groups, their values generally remained within the normal reference ranges. Therefore, these results may not indicate clinically evident organ dysfunction but rather reflect subtle systemic alterations associated with chronic inflammation and OS in COPD. In this context, such changes should be interpreted as supportive findings that accompany the impaired oxidative/antioxidant balance rather than as markers of direct hepatic, renal, or tissue injury. Disruptions in the thiol–disulfide balance represent not only a marker of OS but also a dynamic system involved in inflammation and tissue destruction processes [30]. It has been reported that impairment of the thiol/disulfide balance in COPD may exacerbate airway inflammation and emphysema [9,31]. The mechanisms underlying this imbalance may involve excessive ROS generation from activated neutrophils and macrophages, protease–antiprotease imbalance, and mitochondrial dysfunction, all of which contribute to persistent airway inflammation and progressive tissue damage [32].

From a clinical perspective, these findings highlight potential therapeutic implications. Interventions aimed at restoring redox homeostasis—such as antioxidant supplementation, thiol-containing agents (e.g., N-acetylcysteine, erdosteine), and lifestyle modifications including smoking cessation—could help mitigate OS and inflammation in COPD [33]. Furthermore, targeted therapies modulating oxidative pathways may provide additional benefit in slowing disease progression. Future studies should evaluate whether monitoring and modifying thiol/disulfide balance translates into improved clinical outcomes in COPD patients [25].

This study has several limitations. First, the sample size is relatively small, which may limit the statistical power and generalizability of the findings. Second, the cross-sectional design precludes evaluation of longitudinal changes in OS and thiol/disulfide homeostasis over time. Thiol/disulfide balance represents a potential biomarker in COPD, but further validation is required before it can be applied in clinical practice.

## 5. Conclusions

In conclusion, this study indicates the assessment of the thiol/disulfide balance may be clinically useful in determining disease severity and monitoring treatment responses in patients with COPD. These findings also emphasize the importance of OS in COPD and may contribute to the rationale for developing antioxidant-based therapeutic strategies. However, given the pilot nature of this study and the complex role of this factor in COPD, further large-scale and longitudinal studies are warranted before thiol/disulfide parameters can be established as reliable biomarkers or therapeutic targets.

## Figures and Tables

**Table 1:** Demographic features of the groups.

		Control (*n* = 34)		COPD (*n* = 34)	
		*n*	%	*n*	%
**Gender**	**Female**	6	17.6	6	17.6
	**Male**	28	82.4	28	82.4
**Smoking status**	**Smoker**	12	35.3	30	88.2
	**Non-smoker**	22	64.7	4	11.8
**Comorbidities**	**None**	20	58.8	7	18.4
	**Coronary artery disease**	5	14.7	8	21.1
	**Hypertension**	9	26.5	10	26.3
	**Atrial fibrillation**	0	0.0	1	2.6
	**Diabetes Mellitus**	0	0.0	5	13.2
	**Hyperlipidemia**	0	0.0	7	18.4
GOLD * stages	**Stage 1**	0	0.0	11	34.4
	**Stage 2**	0	0.0	9	26.5
	**Stage 3**	0	0.0	9	26.5
	**Stage 4**	0	0.0	5	14.7

* Global Initiative for Chronic Obstructive Lung Disease.

**Table 2:** Comparison results on laboratory parameters, age and body mass index in the control and COPD groups.

	Groups				*p*-Value
	Control		COPD		
	*n* = 34		*n* = 34		
***Demographic findings***					
**Age (years)**	68.3	5.6	67.1	8.0	0.476
**BMI (kg/m^2^)**	25.8	5.1	25.2	4.4	0.578
***Hematological results***					
**WBC (10^3^/L)**	7.6	(5.4–9.4)	8.7	(6.9–11.6)	0.017 **
**NEU (10^3^/L)**	3.6	(2.8–5.1)	5.1	(4.2–7.5)	0.000 **
**NEU (%)**	61.0	(45–70)	63.5	(55–70)	0.354
**LYMPH (10^3^/L)**	2.0	(1.3–2.6)	1.9	(1.6–2.8)	0.523
**LYMPH (%)**	22.5	(15–38)	23.5	(18–30)	0.787
**MONO (10^3^/L)**	0.3	(0.2–0.5)	0.7	(0.5–0.8)	<0.0001 **
**MONO (%)**	5.4	1.5	7.4	2.4	0.000 *
**EOS (10^3^/L)**	0.3	(0.2–0.4)	0.2	(0.1–0.3)	0.019 **
**EOS (%)**	4.0	(3–5)	2.5	(1–4)	0.002 **
**PLT (10^3^/L)**	281.0	(165–360)	265.0	(219–316)	0.956
**MPV (fL)**	9.3	0.92	10.0	0.99	0.002 *
**Hb (g/dL)**	14.1	(12.6–15.4)	14.0	(13–15)	0.446
***Biochemical results***					
**CRP (mg/L)**	3.0	(2–4)	4.0	(2–7.4)	0.229
**Urea (mg/dL)**	14.5	(12–17)	15.0	(14–18)	0.082
**Creatinine (mg/dL)**	0.9	(0.8–1)	0.8	(0.7–0.9)	0.015 **
**ALT (IU/L)**	25.5	(18–30)	16.5	(14–21)	0.000 **
**AST (IU/L)**	15.5	(12–20)	19.0	(15–20)	0.025 **
**LDH (IU/L)**	167.0	(148–190)	210.0	(183–244)	0.000 **

* Significant difference at <0.05 level according independent *t*-test, Means and Standart deviations (SD) are presented ** Significant difference at <0.05 level according to Mann-Whitney U test, Medians are presented and 25p–75p are shown in parentheses.

**Table 3:** Comparison of oxidative stress parameters between patients and control group.

Oxidative Stress Markers	Groups				*p*-Value
	Control		COPD		
	*n* = 34		*n* = 34		
**Total Thiol (µmol/L)**	462.9	35.2	391.9	26.5	<0.0001 *
**Native Thiol (µmol/L)**	428.3	36.0	351.5	28.3	<0.0001 *
**Disulfide**	16.8	(12.5–21.5)	19.3	(16.6–22.1)	0.048 **
**Disulfide/Native Thiol (%)**	3.97	(2.7–5.1)	5.43	(4.8–6.6)	0.000 **
**Disulfide/Total Thiol (%)**	3.75	1.37	5.18	1.25	<0.0001 *
**Native Thiol/Total Thiol (%)**	92.5	2.7	89.6	2.5	<0.0001 *
**TAS**	1.51	0.30	1.22	0.18	<0.0001 *
**TOS**	5.35	1.26	7.69	0.72	<0.0001 *
**OSI**	0.37	0.11	0.64	0.09	<0.0001 *

* Significant difference at <0.05 level according independent *t*-test, Means and Standart deviations (SD) are presented ** Significant difference at <0.05 level according to Mann-Whitney U test, Medians are presented and 25p–75p are shown in parentheses.

**Table 4:** Comparison of thiol–disulfide homeostasis and oxidative stress parameters across GOLD stages in COPD patients.

	GOLD Criteria																			
	GOLD 1					GOLD 2					GOLD 3					GOLD 4				
	*n*	Mean	SD	Median	IQR	*n*	Mean	SD	Median	IQR	*n*	Mean	SD	Median	IQR	*n*	Mean	SD	Median	IQR
**Total Thiol (µmol/L)**	11	398.7	25.0	409.4	34.4	9	390.6	31.5	384.6	57.2	9	389.1	28.7	396.3	42.0	5	384.5	19.4	375.2	27.8
**Native Thiol (µmol/L)**	11	354.6	27.8	362.0	46.7	9	351.5	33.7	344.3	59.6	9	348.8	32.4	349.1	40.8	5	349.5	16.7	342.1	29.5
**Disulfide**	11	22.1	6.0	21.7	9.1	9	19.6	2.6	20.2	3.0	9	20.2	5.3	18.7	7.0	5	17.5	2.6	16.6	3.0
**Disulfide/Native Thiol (%)**	11	6.30	2.03	5.90	3.02	9	5.64	1.06	5.90	1.60	9	5.88	1.83	5.41	2.48	5	5.01	0.66	4.90	0.81
**Disulfide/Total Thiol (%)**	11	5.55	1.55	5.27	2.38	9	5.05	0.86	5.28	1.29	9	5.22	1.46	4.88	1.96	5	4.55	0.54	4.47	0.67
**TAS**	11	1.20	0.21	1.19	0.21	9	1.32	0.18	1.28	0.21	9	1.16	0.18	1.21	0.25	5	1.19	0.04	1.19	0.06
**TOS**	11	7.53	0.59	7.50	1.08	9	8.01	0.65	8.14	1.08	9	7.74	0.65	7.60	0.75	5	7.34	1.12	6.69	2.02
**OSI**	11	0.64	0.09	0.66	0.14	9	0.62	0.11	0.60	0.17	9	0.68	0.09	0.67	0.12	5	0.62	0.08	0.59	0.14

**Table 5:** Correlation analysis findings between pulmonary function test results and oxidative stress markers.

		FEV1 (mL)	FEV1 (%)	FVC (mL)	FVC (%)	FEV1/FVC (%)
		*n* = 34				
**Total Thiol (µmol/L)**	**r**	0.185	0.090	0.088	0.047	0.183
	***p*** **-Value**	0.294	0.613	0.619	0.791	0.301
**Native Thiol (µmol/L)**	**r**	0.057	0.008	0.011	−0.031	0.121
	***p*** **-Value**	0.747	0.966	0.949	0.862	0.497
**Disulfide**	**r**	0.213	0.229	0.214	0.225	0.150
	***p*** **-Value**	0.226	0.193	0.225	0.201	0.398
**Disulfide/Total Thiol (%)**	**r**	0.116	0.198	0.186	0.211	0.090
	***p*** **-Value**	0.513	0.263	0.293	0.231	0.613
**Disulfide/Native Thiol (%)**	**r**	0.121	0.200	0.185	0.214	0.089
	***p*** **-Value**	0.495	0.257	0.295	0.223	0.615
**Native Thiol/Total Thiol (%)**	**r**	−0.120	−0.198	−0.187	−0.212	−0.090
	***p*** **-Value**	0.499	0.261	0.291	0.229	0.611
**TAS**	**r**	0.056	0.056	0.066	0.018	0.074
	***p*** **-Value**	0.752	0.753	0.711	0.921	0.677
**TOS**	**r**	−0.166	−0.059	−0.197	−0.063	0.015
	***p*** **-Value**	0.348	0.741	0.264	0.725	0.934
**OSI**	**r**	−0.183	−0.093	−0.237	−0.091	0.014
	***p*** **-Value**	0.299	0.601	0.178	0.611	0.939

**Table 6:** Diagnostic performance of oxidative stress parameters for COPD.

	Cut-off	Sensitivity	Specificity	PPV	NPV	AUC (95% CI)		*p*-Value
**Total Thiol (µmol/L)**	≤425.2	94.1	91.2	91.4	93.9	0.952	(0.871–0.989)	<0.0001
**Native Thiol (µmol/L)**	≤394.5	97.1	94.1	94.3	97.0	0.962	(0.886–0.994)	<0.0001
**Disulfide**	>15.5	91.2	44.1	62.0	83.3	0.640	(0.514–0.753)	0.044
**TAS**	≤1.3	79.4	70.6	73.0	77.4	0.796	(0.681–0.884)	<0.0001
**TOS**	>6.2	100	82.4	85.0	100.0	0.948	(0.866–0.987)	<0.0001
**OSI**	>0.49	97.1	91.2	91.7	96.9	0.977	(0.908–0.998)	<0.0001

AUC: Area under curve CI: Confidence interval.

**Table 7:** Results of logistic regression analyses.

Variable	β	SE	Wald X^2^	*p*	OR	OR (95% CI)
**Gender (RC: Female)**	−1.301	0.936	1.932	0.165	0.272	(0.04–1.7)
**Smoking status (RC: Current smoking)**	−2.786	0.846	10.854	0.001 *	0.062	(0.01–0.32)
**Comorbidity (RC: Absent)**	0.877	0.663	1.746	0.186	2.403	(0.65–8.82)
**Age (years)**	−0.001	0.044	0.001	0.975	0.999	(0.92–1.09)
**BMI (kg/m^2^)**	0.003	0.066	0.002	0.967	1.003	(0.88–1.14)

* Significance at <0.05 level. OR: Odds ratio, CI: Confidence interval, RC: Reference category. *n* = 68.

## Data Availability

The data supporting the findings of this study are available from the corresponding author upon reasonable request.
